# Precision treatment and frontier advances of glycyrrhizin preparations in drug-induced liver injury (DILI)

**DOI:** 10.3389/fphar.2026.1890794

**Published:** 2026-08-10

**Authors:** Dianya Qiu, Zixuan Gao, Lu Zhang, Tianyu Ma, Xin Wei, Ziyu Zhang, Weihua Cao, Xinxin Li, Shiyu Wang, Wen Deng, Yaqin Zhang, Linmei Yao, Shuojie Wang, Yuyong Jiang, Yao Xie, Minghui Li

**Affiliations:** 1 Department of Hepatology Division 2, Beijing Ditan Hospital, Capital Medical University, Beijing, China; 2 Clinical Cure and Immunology Joint Laboratory for Clinical Medicine, HBV Infection, Capital Medical University, Beijing, China; 3 Department of Hepatology Division 2, Peking University Ditan Teaching Hospital, Beijing, China

**Keywords:** Drug-induced liver injury (DILI), evidence-based medicine, glycyrrhizin preparations, gut-liver axis, precision treatment

## Abstract

Drug-induced liver injury (DILI) is an important drug-induced disease in clinical practice, with a complex pathogenesis and challenges in diagnosis and treatment. Glycyrrhizin preparations are a class of hepatoprotective agents derived from the traditional Chinese medicinal plant Glycyrrhiza uralensis, and have shown significant efficacy in the treatment of DILI due to their multi-target and multi-pathway pharmacological effects. Currently, they have been recommended as core therapeutic drugs for DILI in multiple domestic and international guidelines. This review delves into the advanced evidence-based medical evidence for the treatment of DILI with glycyrrhizin preparations and their recommended status in various guidelines. It also systematically expounds the precise treatment strategies of glycyrrhizin preparations based on DILI classification, severity, and special populations, and elaborately analyzes their cutting-edge mechanisms of action such as regulation of the gut-liver axis and epigenetic modifications. It discusses the challenges in clinical application, such as adverse reactions and predictive markers of efficacy, and their optimization solutions, and looks forward to future research directions. This article aims to promote the precise application of glycyrrhizin preparations in the treatment of DILI, providing practical diagnostic and therapeutic references for clinicians.

## Graphical Abstract

**Figure:**
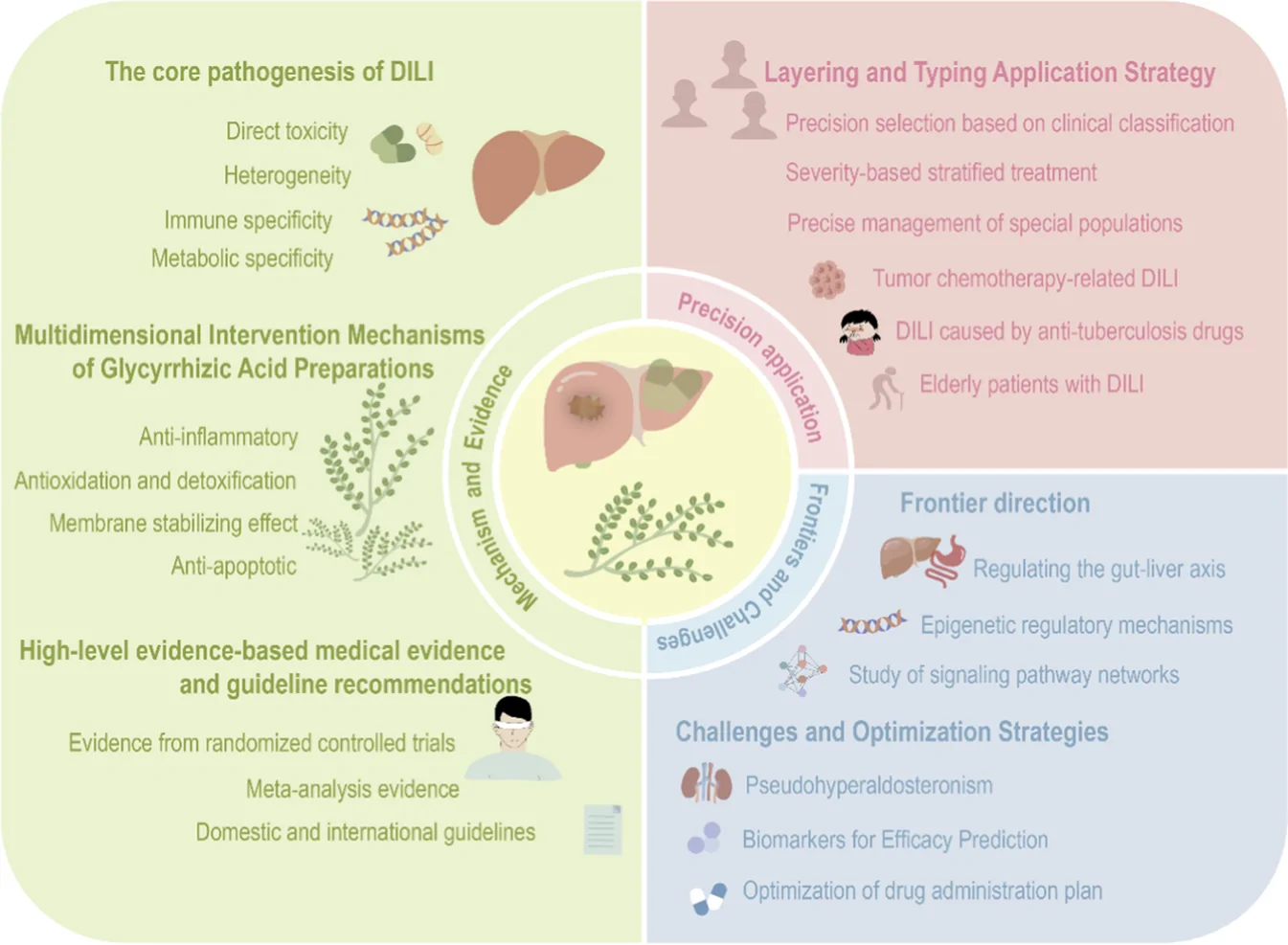
This figure summarizes the overall framework and core viewpoints of the present review, covering the clinical classification and pathogenesis of drug-induced liver injury (DILI), the multi-target hepatoprotective mechanisms of glycyrrhizin preparations (including anti-inflammatory, antioxidant, membrane-stabilizing and anti-apoptotic effects), as well as individualized therapeutic strategies built on evidence-based medicine and precision medicine principles (i.e., stratified management based on DILI subtypes, disease severity and special populations). The figure further highlights cutting-edge mechanisms such as gut–liver axis regulation and epigenetic modification, which provide theoretical support for precision clinical medication. Figure was created by the authors.

## 1 Introduction

Drug-induced liver injury (DILI) refers to liver damage caused by chemically synthesized drugs, biologics, proprietary Chinese medicines (classified as prescription or over-the-counter medications), traditional Chinese medicinal materials, natural medicines, health supplements, dietary supplements, or their metabolites, excipients, contaminants, and impurities (Technology Committee on DILI Prevention and Management and Chinese Medical Biotechnology Association, 2023). Globally, the reported annual incidence of DILI ranges from approximately 1.0–20.0 cases per 100,000 population across different geographic regions and study periods. However, in developing countries such as China, due to factors including a large population base, extensive use of medications, widespread application of traditional medicines, and a relatively lenient regulatory system, DILI has become an increasingly severe public health issue. According to a nationwide epidemiological study conducted between 2012 and 2014 in mainland China, the annual incidence of DILI was estimated to be 23.8 cases per 100,000 population, which is significantly higher than that reported in many developed countries (Ou et al., 2015; Study of Drug Induced Liver Disea se of Chinese Medical Association, 2015; Ahmad and Odin, 2017; Shen et al., 2019; Sandhu and Navarro, 2020; Wei et al., 2024). DILI is now one of the leading causes of acute liver failure (ALF), with a mortality rate as high as 50% in ALF cases caused by DILI, imposing a substantial burden on healthcare systems (Katarey and Verma, 2016; Lee, 2003; Hosack et al., 2023).

The clinical phenotypes of DILI are highly heterogeneous. Based on the primary injured cells and biochemical characteristics, DILI is classified into three types: hepatocellular injury type, cholestatic type, and mixed type (Bénichou, 1990). Among these, hepatocellular injury-type DILI is the most common and is more prone to progress to ALF with higher mortality rates (Shen et al., 2019; Shmeleva and Mukhina, 1974). Diagnosing DILI remains challenging, relying predominantly on exclusion criteria. The Roussel Uclaf Causality Assessment Method (RUCAM) scale is widely adopted internationally as a key diagnostic tool, though it still has limitations. Consequently, clinical expertise plays a crucial role in the diagnostic process (Teschke et al., 2022; Andrade et al., 2019; Danan and Teschke, 2015). In terms of treatment, aside from immediate discontinuation of suspected drugs and supportive care, there remains a lack of specific therapeutic agents. These limitations in treatment options highlight the urgent need for improved clinical management of DILI (Li et al., 2021; Björnsson, 2021).

Glycyrrhizin preparations, a class of hepatoprotective agents derived from the traditional Chinese medicinal herb Glycyrrhiza uralensis and comprising clinically used formulations such as magnesium isoglycyrrhizinate, diammonium glycyrrhizinate, and compound glycyrrhizin, have become a cornerstone in DILI treatment after decades of clinical practice and scientific research (Tian et al., 2021; Bi et al., 2023). These preparations exert hepatoprotective effects through multifaceted mechanisms, including anti-inflammatory, antioxidant, and anti-apoptotic actions (Li et al., 2014). With the advancement of precision medicine concepts and molecular biology technologies, the application of glycyrrhizin preparations in DILI therapy is transitioning from a traditional “one-size-fits-all” approach to precision strategies based on biomarkers, disease subtypes, and individual characteristics.

**FIGURE 1:**
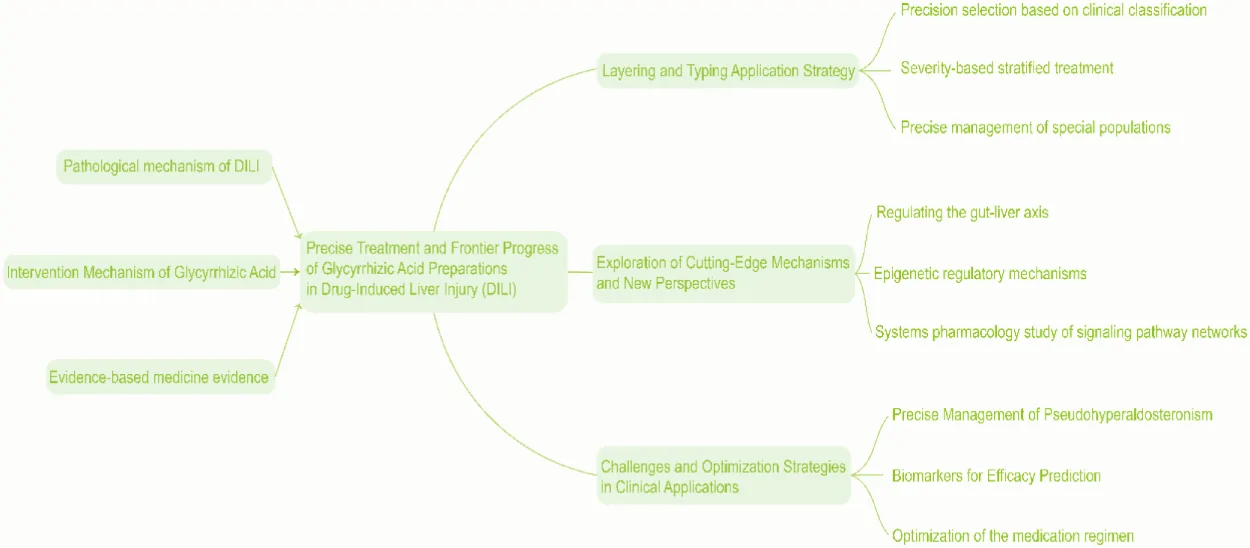
Mind Map. This figure illustrates the overall framework of the review. It begins with the core pathogenic mechanisms of drug-induced liver injury (DILI) and introduces the therapeutic targets of glycyrrhizin preparations. It then summarizes high-level evidence from randomized controlled trials, meta-analyses, and clinical practice guidelines. Based on this evidence, a precision treatment strategy is proposed according to DILI clinical phenotype, disease severity, and special patient populations. Finally, the figure highlights emerging mechanisms, including the gut–liver axis, epigenetic regulation, and systems pharmacology, as well as current challenges and future directions for optimizing the clinical application of glycyrrhizin preparations. Figure was created by the authors.

Although several recent reviews have summarized the pharmacological effects of glycyrrhizin preparations and their applications in liver diseases, and domestic and international guidelines and expert consensuses have provided standardized recommendations for the diagnosis and management of drug-induced liver injury (DILI), most existing studies have focused on a single dimension—namely, clinical efficacy, mechanisms of action, or therapeutic recommendations—and there remains a paucity of reviews that systematically integrate high-level evidence-based medicine, precision therapeutic strategies, and emerging mechanistic insights. To address this gap, the present review, grounded in precision medicine, first systematically consolidates high-level evidence from randomized controlled trials, meta-analyses, and domestic and international guidelines to evaluate the clinical value of glycyrrhizin preparations as a foundational therapeutic agent for DILI. Subsequently, we construct evidence-based precision therapeutic strategies tailored to different DILI subtypes, severity grades, and special populations. Finally, we summarize emerging mechanistic studies—including gut-liver axis regulation, epigenetic modification, and systems pharmacology—and discuss their potential value in future precision medicine. Compared with previous reviews that primarily focused on pharmacological mechanisms, clinical efficacy, or guideline recommendations, the present review further integrates evidence-based medicine, precision therapeutic strategies, and frontier mechanistic research to establish a comprehensive “evidence-strategy-mechanism” precision management framework for glycyrrhizin preparations in the treatment of DILI, thereby providing a more systematic reference for clinical decision-making and future investigations (Figure 1).

## 2 Pathological mechanisms of DILI and intervention targets of glycyrrhizin preparations

### 2.1 Core pathogenic mechanisms of DILI

The pathogenesis of DILI is complex and involves two primary pathways: direct toxicity and idiosyncratic reactions.

#### 2.1.1 Direct toxicity of DILI

The direct toxicity of DILI is typically dose-dependent and closely associated with the inherent hepatotoxicity of drugs or their metabolites (Ma et al., 2023). A representative example is liver injury caused by acetaminophen (APAP) overdose (Cavalieri and D’Agostino, 2017). APAP undergoes oxidative metabolism in the body via the cytochrome P450 (CYP450) enzyme system, generating the reactive intermediate metabolite N-acetyl-p-benzoquinone imine (NAPQI). At physiological or therapeutic doses, glutathione (GSH) in hepatocytes specifically and covalently binds to NAPQI, converting it into non-toxic metabolites for elimination, thereby achieving effective detoxification. However, during APAP overdose, excessive activation of the CYP450 system leads to overproduction of NAPQI, rapidly depleting the limited intracellular GSH reserves and rendering the liver incapable of further NAPQI clearance. Excess NAPQI covalently binds to hepatic proteins, forming NAPQI-protein adducts that disrupt protein structure and function, subsequently inducing severe oxidative stress and mitochondrial dysfunction. This process is accompanied by collapse of mitochondrial membrane potential, impaired ATP synthesis, and massive reactive oxygen species (ROS) production. ROS further amplifies oxidative damage, ultimately leading to hepatocyte necrosis (Cai et al., 2022).

#### 2.1.2 Idiosyncratic DILI

Idiosyncratic DILI is generally independent of drug dosage. Its onset arises from the combined effects of genetic susceptibility, immune responses and environmental factors, and is also modulated by individual drug metabolic profiles (Asif et al., 2024). This type of injury can be categorized into two subtypes: immune-mediated and metabolism-specificDILI (Chalasani et al., 2021). Immune-mediated DILI involves drug metabolites acting as haptens that bind to endogenous hepatic proteins, forming novel antigens. These antigens are processed by antigen-presenting cells (APCs), activating immune cells such as T lymphocytes (CD4^+^ and CD8^+^ T cells) and eosinophils, which trigger adaptive immune responses and hepatocyte damage (Asif et al., 2024; Uetrecht, 2025; Liu et al., 2021). Studies have demonstrated that specific human leukocyte antigen (HLA) genotypes are significantly associated with the risk of DILI induced by certain drugs (e.g., flucloxacillin), indicating that genetic susceptibility serves as a critical basis for the development of immune-mediated DILI. In addition, innate immune activation triggered by drugs or their metabolites can further disrupt host immune tolerance, sustain the activation of drug-specific T cells, and ultimately amplify adaptive immune responses to provoke hepatocellular injury (Daly et al., 2009). Metabolism-specific DILI primarily originates from genetic polymorphisms in drug-metabolizing enzymes. Genetic variations among individuals lead to differences in enzyme activity, resulting in abnormal accumulation of reactive metabolites or impaired detoxification (Singh et al., 2023). Among these enzymes, the CYP450 superfamily—a major system in drug metabolism—is a common cause of metabolism-specific DILI due to its genetic diversity (Maddrey, 2005; Cox et al., 2020). However, this mechanism is not exclusive to CYP450; other metabolizing enzymes with polymorphisms may also induce hepatotoxicity. For example, the hepatotoxicity of isoniazid strongly correlates with N-acetyltransferase 2 (NAT2) acetylator phenotypes, where slow acetylators exhibit higher risks of liver injury (Huang, 2014).

Notably, accumulating evidence indicates that idiosyncratic DILI does not arise from a single mechanism; instead, it represents a complex disease that develops under the combined interplay of genetic susceptibility, immune responses and environmental factors. Beyond polymorphisms in HLA and drug-metabolizing enzyme genes, other genetic variants related to drug transporters and inflammatory regulation may also modify an individual’s vulnerability to drug-induced liver injury. Meanwhile, environmental triggers including infection, age, gender, alcohol consumption, pre-existing liver disease, concomitant medication use and gut microbiota can further alter DILI risk and clinical manifestations by reshaping drug metabolism, immune homeostasis or the inflammatory microenvironment. Accordingly, a consensus has been established that idiosyncratic DILI results from the synergistic interaction of genetic background, immune dysregulation and environmental exposures, and its intricate multifactorial pathogenesis largely accounts for the ongoing challenges in its prediction and prevention (Andrade et al., 2019; Chalasani et al., 2021; Uetrecht, 2019; Kaplowitz, 2005).

**FIGURE 2:**
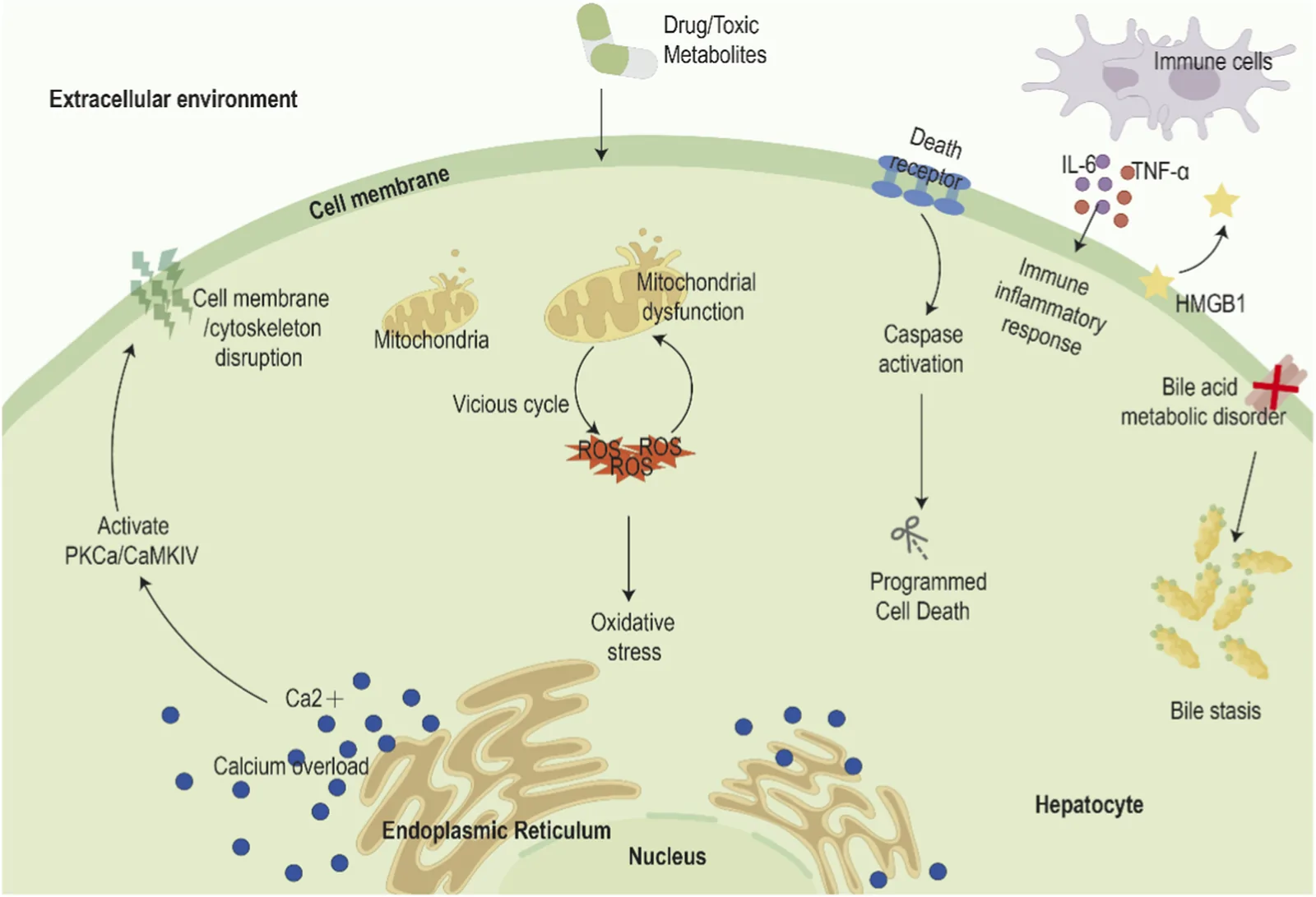
Common Convergent Pathways in DILI Pathogenesis. All subtypes of DILI (direct hepatotoxicity or idiosyncratic reactions) ultimately converge on shared pathological cascades, including mitochondrial dysfunction, oxidative stress, calcium overload, death receptor-mediated hepatocellular apoptosis, amplified immune inflammation, and disrupted bile acid metabolism. Crosstalk among these pathways synergistically drives hepatocellular necrosis and apoptosis. Red arrows in the figure denote injury amplification or positive feedback loops. Figure was created by the authors.

Both mechanisms ultimately converge on the following pathways to induce liver injury (Figure 2): 1. Mitochondrial dysfunction and oxidative stress, driving excessive reactive oxygen species (ROS) production, which exacerbates mitochondrial damage in a vicious cycle; 2. Calcium homeostasis imbalance (e.g., via the PLCγ1–IP_3_R–SOC signaling pathway), leading to calcium overload and activation of calcium-dependent molecules (e.g., PKCα, CaMKIV), thereby disrupting membrane integrity and cytoskeletal stability; 3. Death receptor-mediated apoptosis pathway activation, initiating programmed cell death; 4. Amplified immune-inflammatory responses, mediated by damage-associated molecular patterns (e.g., HMGB1) and immune pathway activation, intensifying inflammatory damage; 5. Bile acid metabolism dysregulation and cholestasis, caused by dysfunction of bile acid transporters or metabolism-related molecules, resulting in toxic bile acid accumulation (Allison et al., 2023; Zhao et al., 2017).

### 2.2 Multidimensional therapeutic mechanisms of glycyrrhizin preparations

**FIGURE 3:**
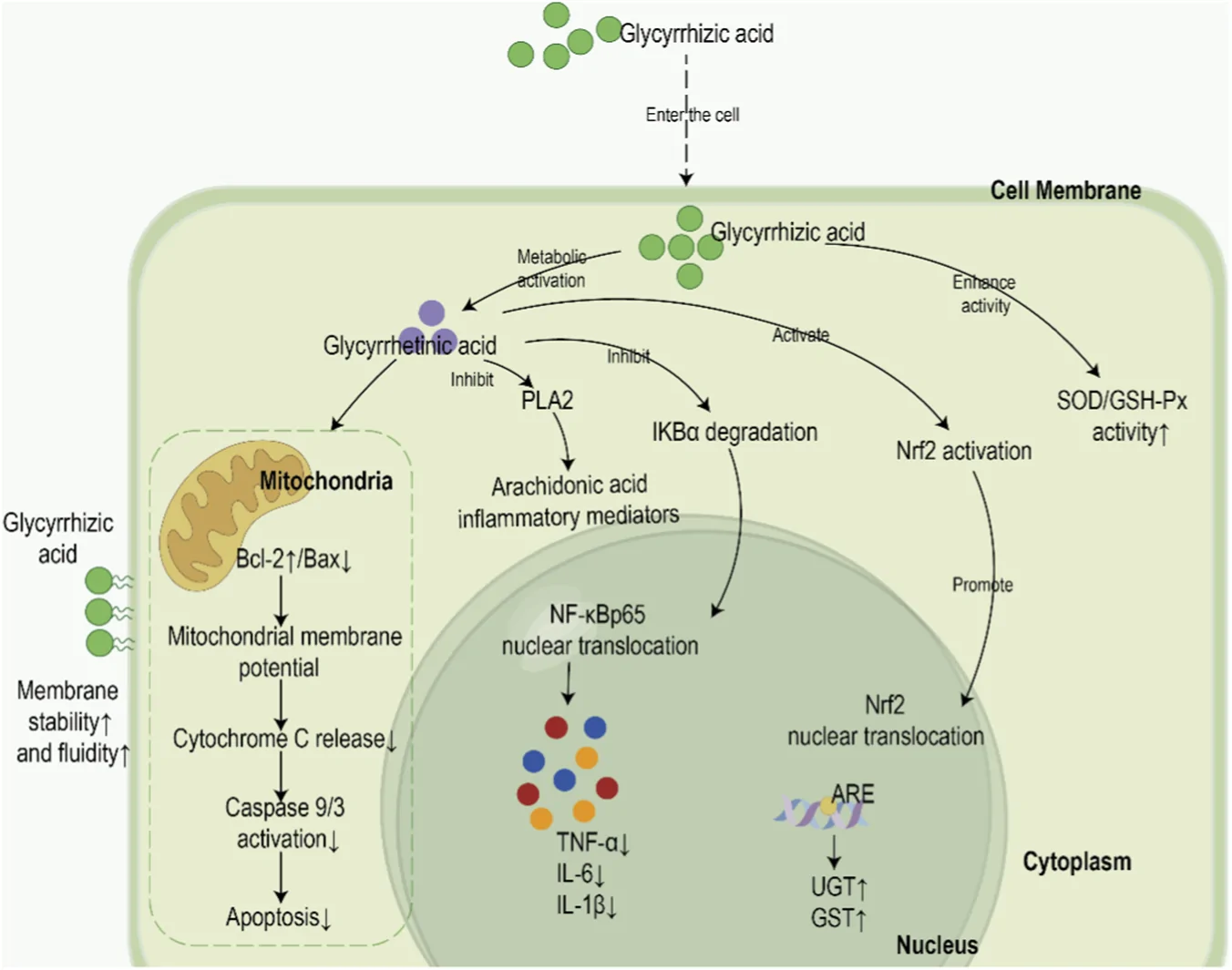
Hepatoprotective Mechanisms of Glycyrrhizin Preparations in DILI. Glycyrrhizin preparations exert hepatoprotective effects, primarily through their active metabolite glycyrrhetinic acid (GA), which acts via multiple mechanisms, including anti-inflammation, antioxidation/detoxification, membrane stabilization, and anti-apoptosis. Its anti-inflammatory activity mainly targets the PLA2 and NF-κB signaling pathways; antioxidant capacity relies on the Nrf2/ARE axis to boost endogenous antioxidant systems; membrane-stabilizing effects preserve the integrity of hepatocyte membranes; and anti-apoptotic function is achieved primarily through regulating the Bcl-2/Bax ratio and caspase cascades. Figure was created by the authors.

The initiation and progression of drug-induced liver injury (DILI) involve multiple interconnected pathological processes, including inflammatory response, oxidative stress, mitochondrial dysfunction, cellular apoptosis and cell membrane damage. These pathological cascades interact with and amplify one another, collectively driving hepatocellular injury and disease deterioration. Accordingly, comprehensive intervention targeting multiple critical pathological links is regarded as an important strategy to improve therapeutic outcomes of DILI. Accumulating basic research has demonstrated that glycyrrhizin preparations possess multi-target and multi-dimensional pharmacological profiles, which can interfere with each of the above core pathological processes to exert synergistic hepatoprotective effects (Figure 3) (Andrade et al., 2019).

#### 2.2.1 Anti-inflammatory effects

Inflammatory response is one of the core pathological mechanisms underlying the initiation and progression of DILI. Drugs and their active metabolites trigger the release of damage-associated molecular patterns (DAMPs), which activate Kupffer cells and other immune cells. This process further stimulates inflammatory signaling cascades including NF-κB, driving robust secretion of pro-inflammatory cytokines such as TNF-α, IL-1β and IL-6, and consequently amplifying inflammatory reactions within hepatic tissue. Accordingly, suppression of inflammatory cascades has emerged as a vital therapeutic strategy for DILI, and anti-inflammatory activity constitutes one of the most central pharmacological mechanisms of glycyrrhizin preparations (Fujisawa et al., 2000; Mori et al., 1990). The active metabolite glycyrrhetinic acid (GA) potently inhibits phospholipase A2 (PLA2) activity, reducing the release of arachidonic acid from membrane phospholipids and thereby blocking the synthesis of inflammatory mediators such as prostaglandins and leukotrienes (Fujisawa et al., 2000; Honda et al., 2012; Takei et al., 2006). More importantly, GA exerts profound suppression on the NF-κB signaling pathway. At the molecular level, GA inhibits the phosphorylation and degradation of IκBα protein, preventing nuclear translocation of the NF-κB p65 subunit, which subsequently downregulates the transcription of key pro-inflammatory cytokines including TNF-α, IL-6, and IL-1β (Honda et al., 2012; Sch et al., 2009). Studies confirm that GA inhibits NF-κB p65 nuclear translocation in a dose-dependent manner (Honda et al., 2012).

#### 2.2.2 Antioxidant and detoxification effects

Oxidative stress represents one of the earliest and most pivotal pathological events activated during the progression of DILI. Drugs and their active metabolites trigger massive overproduction of reactive oxygen species (ROS), which in turn induce lipid peroxidation, DNA damage and oxidative modification of proteins. Meanwhile, these toxicants impair the endogenous antioxidant defense system, exacerbating hepatocellular injury. Accordingly, boosting antioxidant capacity and facilitating the clearance of toxic metabolites constitute key therapeutic strategies for DILI. Glycyrrhizin preparations exert multi-layered protective effects against oxidative damage and xenobiotic toxicity (Villanueva-Paz et al., 2021; Grattagliano et al., 2009; Gumpricht et al., 2005; Wu et al., 2021). They significantly enhance the activity of endogenous antioxidant enzymes such as superoxide dismutase (SOD), catalase, and glutathione peroxidase (GSH-Px), improving the body’s capacity to scavenge oxygen free radicals and suppress lipid peroxidation (Wang Q. et al., 2022; Shi et al., 2021). Additionally, GA activates the Nrf2-Keap1 pathway, promoting antioxidant response element (ARE)-driven gene transcription to upregulate Phase II metabolic enzymes (e.g., UDP-glucuronosyltransferase, glutathione S-transferase), thereby enhancing detoxification of drug metabolites (Chu et al., 2020; Zhu et al., 2021; Li et al., 2018; Li et al., 2016; Yang et al., 2001). Notably, nano-gels combining glycyrrhizin with the antioxidant quercetin exhibit superior antioxidant activity and remarkable hepatoprotective efficacy in acute liver injury models (Zhao et al., 2021).

#### 2.2.3 Maintenance of mitochondrial functional homeostasis

Mitochondrial dysfunction is recognized as one of the shared terminal pathological mechanisms of DILI, and also serves as a critical link between oxidative stress and cellular apoptosis. Drugs or their active metabolites trigger the collapse of mitochondrial membrane potential, reduced ATP production, and aberrant opening of the mitochondrial permeability transition pore (mPTP). These alterations further drive sustained ROS accumulation and cytochrome C release, ultimately inducing hepatocellular death (Labbe et al., 2008; Jaeschke et al., 2012). Existing studies have demonstrated that glycyrrhizin preparations ameliorate mitochondrial ultrastructure, stabilize mitochondrial membrane potential, suppress ROS overproduction, boost ATP synthesis, and block mPTP opening. Such effects alleviate drug-triggered mitochondrial damage and lay an essential foundation for the subsequent anti-apoptotic activity of glycyrrhizin (Gumpricht et al., 2005; Wu et al., 2021).

#### 2.2.4 Membrane-stabilizing effects

Cell membrane injury constitutes one of the prominent pathological alterations in DILI. Drugs and their active metabolites disrupt the lipid bilayer structure of hepatocyte membranes, increase membrane permeability, and trigger the leakage of intracellular enzymes followed by cellular damage. Glycyrrhizin stabilizes the structural integrity of hepatocyte membranes and mitigates direct hepatocellular injury induced by toxic drug derivatives. GA possesses an amphiphilic structure underpins this mechanism. The hydrophobic region embeds into the phospholipid bilayer, while the hydrophilic domain interacts with the membrane surface. This insertion reinforces membrane stability and fluidity, reducing structural disruption by drugs or metabolites and preventing leakage of intracellular enzymes like transaminases (Chu et al., 2020; Zhu et al., 2021). Biophysical studies reveal that GA maintains the ordered arrangement of membrane lipids and reduces membrane permeability—an essential feature for counteracting lipophilic drug-induced membrane damage (Nakamura et al., 1985; Selyutina and Polyakov, 2019).

#### 2.2.5 Anti-apoptotic effects

Sustained oxidative stress and mitochondrial dysfunction ultimately trigger hepatocellular apoptosis, representing one of the key terminal pathological cascades in DILI. Glycyrrhizin preparations exert protective effects by regulating apoptosis-related signaling pathways. Specifically, GA upregulates anti-apoptotic proteins (e.g., Bcl-2), downregulates pro-apoptotic proteins (e.g., Bax), and optimizes the Bcl-2/Bax ratio, stabilizing mitochondrial membrane potential and inhibiting cytochrome C release (Wu et al., 2020). GA further blocks apoptotic signaling by suppressing the activation of caspase-9 and caspase-3 (Wu et al., 2020; Liang et al., 2015; Guo et al., 2013). Experimental studies demonstrate that magnesium isoglycyrrhizinate alleviates APAP-induced apoptotic liver injury. Crucially, APAP hepatotoxicity involves caspase-3 activation, providing insights into glycyrrhizin’s protective mechanisms (Xia et al., 2025; Zhang et al., 2023; Abdel-Bakky et al., 2021).

## 3 High-level evidence-based medical evidence and guideline recommendations

To enhance the systematic and logical synthesis of evidence, this chapter focuses on high-level evidence to provide a comprehensive overview of the clinical evidence supporting the use of glycyrrhizin preparations in the treatment of DILI. The evidence included in this chapter was selected based on its level of evidence, study design, and clinical relevance to the use of glycyrrhizin preparations in DILI, with priority given to randomized controlled trials (RCTs), meta-analyses, systematic reviews, and current domestic and international clinical practice guidelines and expert consensus statements. In synthesizing the available evidence, particular attention was paid to study design, sample size, appropriateness of outcome measures, consistency of findings, and clinical applicability, while the conclusions were interpreted in the context of the methodological limitations of individual studies. It should be noted that the currently available evidence remains subject to several limitations, including relatively small sample sizes, uneven geographic distribution of study populations, heterogeneity in outcome measures, and insufficient long-term follow-up data. Therefore, the available evidence should be interpreted with careful consideration of study quality, study limitations, and the specific clinical context, thereby providing an evidence-based foundation for the subsequent discussion of precision therapeutic strategies.

### 3.1 Randomized controlled trial (RCT) evidence

A single-center randomized positive-controlled RCT enrolled 104 patients with acute DILI, who were allocated to the experimental group (80 patients) and control group (24 patients) at a 3:1 ratio. Patients in the experimental group received intravenous infusion of magnesium isoglycyrrhizinate injection 200 mg diluted in 250 mL 10% glucose injection once daily. The control group was administered tiopronin injection 200 mg dissolved in 250 mL 10% glucose injection via intravenous drip once daily. The treatment course lasted 2 weeks for both groups. The results demonstrated that the experimental group achieved significantly higher overall comprehensive response rate at week 2 (77.5%), alanine transaminase (ALT) single-index response rate (74.0%), and aspartate transaminase (AST) single-index response rate (85.6%) compared with the control group (41.7%, 37.5%, 59.1%, all P < 0.05), accompanied by a more prominent reduction in ALT levels. The incidence of adverse reactions was comparable between the two groups (5.0% vs 4.2%, P = 1.00) (Chen et al., 2014). Another randomized, double-blind, multi-dose, positive-controlled multicenter phase II trial recruited 174 patients with acute DILI, who were randomly assigned to three groups at a 1:1:1 ratio: Group A (magnesium isoglycyrrhizinate 100 mg/d diluted in 250 mL 5% glucose solution for once-daily intravenous infusion), Group B (magnesium isoglycyrrhizinate 200 mg/d diluted in 250 mL 5% glucose solution for once-daily intravenous infusion), and Group C (tiopronin 200 mg/d diluted in 250 mL 5% glucose solution for once-daily intravenous infusion). All three groups received a 4-week treatment course. At week 4, the ALT normalization rates in Group A and Group B were significantly superior to that in Group C (84.75% vs 61.02%, P = 0.0029; 85.71% vs 61.02%, P = 0.0037). Logistic regression analysis further revealed that the odds of ALT normalization in Group A and Group B were 3.55 times (OR = 3.55, 95%CI: 1.47–8.57, P = 0.0049) and 3.83 times (OR = 3.83, 95%CI: 1.54–9.55, P = 0.0039) higher than those in Group C, respectively. This study validated that magnesium isoglycyrrhizinate is an effective and safe therapeutic option for acute DILI (Wang et al., 2019). A randomized open-label trial enrolled 80 patients with chronic DILI/herb-induced liver injury (HILI), who were randomly treated with either 48-week tapered methylprednisolone combined with glycyrrhizin or glycyrrhizin monotherapy. The results indicated that combination therapy significantly elevated the sustained biochemical response rate (94.3% vs 71.4%, P = 0.023), shortened the recovery time of biochemical indicators, and alleviated hepatic inflammatory activity and fibrosis compared with glycyrrhizin monotherapy. No severe adverse events were observed during the trial, providing clinical evidence for combination therapeutic regimens in patients with chronic DILI/HILI (Wang J.-B. et al., 2022).

### 3.2 Meta-analysis evidence

Multiple meta-analyses have progressively elucidated the relative efficacy of different glycyrrhizin preparations in treating DILI through diverse comparative frameworks, reflecting an evolving understanding in clinical decision-making. A 2019 meta-analysis covering 24 RCTs with a total of 2,116 patients demonstrated that diammonium glycyrrhizinate yielded a higher overall response rate than other hepatoprotective agents for DILI (RR = 1.20, P < 0.0001), with more pronounced reductions in ALT (WMD = −20.01) and AST (WMD = −64.36), shorter time to biochemical normalization, and comparable safety profiles (Yu et al., 2019). Most primary RCTs included in this meta-analysis adopted diammonium glycyrrhizinate at a daily dose of 150 mg via intravenous infusion or oral administration for a treatment course of 2–4 weeks, yet moderate heterogeneity was detected across individual studies. A network meta-analysis published in 2022 enrolled 64 therapeutic RCTs involving 5,161 patients to systematically compare the relative efficacy of various glycyrrhizin preparations in anti-tuberculosis drug-induced DILI. The original trials incorporated heterogeneous administration regimens for different glycyrrhizin derivatives: magnesium isoglycyrrhizinate was mostly administered intravenously at 100–200 mg daily for 2–4 weeks; diammonium glycyrrhizinate was commonly given orally or intravenously at 150 mg per day; compound glycyrrhizin was available in both oral and intravenous formulations with a standard 2–4 weeks treatment duration. Nevertheless, the network meta-analysis ranked the efficacy of each preparation via indirect comparisons. The outcomes indicated that magnesium isoglycyrrhizinate (MGIG) achieved the highest therapeutic response rate among all glycyrrhizin formulations (RR range: 1.15–1.72), significantly outperforming compound glycyrrhizin, diammonium glycyrrhizinate and other alternatives, rendering it one of the optimal regimens for anti-tuberculosis drug-related DILI (Gong et al., 2022a). A 2025 meta-analysis including 13 RCTs with 1,435 participants systematically evaluated the efficacy and safety of four glycyrrhizin preparations for AT-DILI: magnesium isoglycyrrhizinate (MI), diammonium glycyrrhizinate (DG), compound ammonium glycyrrhizinate (CAG), and compound glycyrrhizin (CG). Given the substantial heterogeneity in administration modalities (formulation, dosage and treatment duration) across the 13 primary RCTs, the core merit of this pooled analysis lies in providing comparative estimates of relative efficacy. The results revealed that MI produced a significantly higher therapeutic response rate than the other three agents (OR = 4.57, 95%CI: 2.93–7.12, P < 0.001), and exhibited superior potency in lowering ALT (MD = −21.63, P < 0.001) and total bilirubin (MD = −11.07, P = 0.03). The efficacy hierarchy was determined as MI > DG ≈ CAG > CG, while no significant intergroup differences were observed in the incidence of adverse reactions (Yang et al., 2025).

### 3.3 Recommendations from domestic and international guidelines

Based on the above evidence from randomized controlled trials and meta-analyses, multiple domestic and international guidelines and expert consensus statements have evaluated the clinical application of glycyrrhizin preparations in the treatment of DILI. Nevertheless, discrepancies exist in the strength of recommendations for glycyrrhizin preparations across different guidelines, attributable to variations in evidence sources, rationale for recommendations and clinical practice contexts. Therefore, this section conducts a comprehensive analysis of the recommendations regarding glycyrrhizin preparations for DILI as well as their clinical implications, with reference to representative domestic and international guidelines (Table 1).

**TABLE 1:** Summary of academic guidelines/consensus recommendations on glycyrrhizin preparations in DILI management.

Guidelines/Consensus	Year of publication	Core position and recommendations	Key insights and features	References
Expert consensus on clinical application of glycyrrhizin preparation in the treatment of liver diseases	2016	Strongly endorses its use for treatment; does not endorse routine use for prevention	First to systematically define the standardized role of these agents in DILI management, clarifying the distinction between therapeutic and prophylactic use	Author Anonymous (2016)
Chinese guideline for diagnosis and management of drug-induced liver injury (2023 version)	2023	A core therapeutic agent, receiving the highest-grade recommendation	Specifically indicates that magnesium isoglycyrrhizinate is currently the only drug with an approved indication for acute DILI, affirming its anti-inflammatory and multi-faceted hepatoprotective mechanisms	(Technology Committee on DILI Prevention and Management and Chinese Medical Biotechnology Association, 2023; Ma et al., 2023)
Drug-induced liver injury: Asia Pacific Association of Study of Liver consensus guidelines	2021	An important therapeutic option, particularly for moderate-to-severe hepatocellular injury	Highlights the need to consider regional prescribing habits in Asia, advising attention to DILI/herb-induced liver injury (HILI) risk and the use of glycyrrhizin preparations in the context of combining traditional and modern medicines	Devarbhavi et al. (2021)
AASLD practice guidance on drug, herbal, and dietary supplement-induced liver injury	2023	No direct recommendation, but its therapeutic principles provide mechanistic support for glycyrrhizin preparations	Provides corroborating rationale from an international perspective, supporting the application logic of anti-inflammatory mechanism-based drugs (including glycyrrhizin preparations) in a broader DILI context	Fontana et al. (2023)
EASL Clinical Practice Guidelines: Drug-induced liver injury	2019	No specific recommendation for glycyrrhizin preparations; emphasizes standardized diagnosis, causality assessment, withdrawal of suspected drugs and evidence-based management of DILI.	Provides internationally accepted diagnostic and management framework, serving as an important benchmark for evaluating future evidence of hepatoprotective therapies	European association for the study of the liver EASL clinical practice guidelines: Drug-Induced liver injury (2019)

Expert consensus on clinical application of glycyrrhizin preparation in the treatment of liver diseases (2016) This consensus systematically established the role of glycyrrhizin preparations in DILI therapy. It strongly recommended (Grade AI) initiating glycyrrhizin-based hepatoprotective and anti-inflammatory treatment after discontinuing the causative drug. However, it explicitly advised against routine prophylactic use to reduce DILI incidence due to insufficient evidence at the time (Author Anonymous, 2016). This consensus statement systematically standardized the therapeutic positioning of glycyrrhizin preparations for DILI for the first time, clearly distinguishing their indications for treatment versus prophylaxis, and providing an essential reference for subsequent domestic clinical practice.

Chinese guideline for diagnosis and management of drug-induced liver injury (2023 version) This guideline designates glycyrrhizin preparations as core therapeutics for DILI. Key recommendations include: Magnesium isoglycyrrhizinate receives the highest recommendation (Class I recommendation, Level A evidence) for acute hepatocellular or mixed-type DILI with significantly elevated ALT, emphasizing its status as the only agent approved for acute DILI indications. Other glycyrrhizin agents (e.g., diammonium glycyrrhizinate, compound glycyrrhizin) are recommended for mild-to-moderate hepatocellular DILI. The guideline underscores their multi-mechanistic hepatoprotective effects, particularly anti-inflammatory actions, in reducing transaminase levels and promoting recovery (Technology Committee on DILI Prevention and Management and Chinese Medical Biotechnology Association, 2023; Ma et al., 2023; Mao, 2024). Overall, Chinese guidelines deliver positive recommendations for glycyrrhizin preparations based on available clinical studies and evidence-based data, with particular emphasis on the clinical value of magnesium isoglycyrrhizinate in acute hepatocellular injury-predominant DILI.

Asian Pacific Association for the Study of the Liver (APASL) Consensus Focusing on Asia-specific practices like herb-chemotherapy combinations, the APASL consensus highlights the need for intensified DILI/HILI monitoring. The APASL consensus places particular emphasis on the epidemiological characteristics of DILI and HILI in Asia, and highlights the necessity of strengthening the identification, monitoring and standardized management of DILI when traditional medicines are combined with modern pharmaceutical agents. With respect to glycyrrhizin preparations, APASL recognizes them as one of the major therapeutic alternatives for DILI with hepatocellular injury pattern, yet stresses that their clinical use should be individualized based on patients’ clinical status and regional medication characteristics (Devarbhavi et al., 2021).

American Association for the Study of Liver Diseases (AASLD) Guidance The 2023 AASLD clinical guidance centers on DILI diagnosis, causality assessment, risk factor identification and clinical management, underscoring that prompt discontinuation of suspected culprit drugs remains the primary intervention for DILI alongside etiology-specific supportive care (Fontana et al., 2023). This guidance offers no explicit recommendations regarding glycyrrhizin preparations, largely due to the scarcity of large-scale, multicenter randomized controlled trials worldwide to validate their routine clinical administration.

In addition, the European Association for the Study of the Liver (EASL) issued the Drug-induced Liver Injury Clinical Practice Guidelines in 2019. This guideline systematically standardizes the diagnostic workflow, causality assessment, severity grading and clinical management of DILI. It emphasizes timely discontinuation of suspected culprit drugs and standardized evaluation using tools such as the RUCAM scale. Although the guideline fails to deliver explicit recommendations for hepatoprotective agents including glycyrrhizin preparations, its established standardized diagnostic and therapeutic framework serves as a cornerstone for international DILI management, and provides a unified reference criterion for evaluating the clinical efficacy of glycyrrhizin preparations and accumulating international evidence-based data in future research (European association for the study of the liver EASL clinical practice guidelines: Drug-Induced liver injury, 2019). Accordingly, EASL has not yet formulated dedicated recommendations for glycyrrhizin preparations; instead, it stresses that all hepatoprotective therapies should be implemented on the basis of standardized diagnosis, timely drug withdrawal and evidence-based medical data.

Overall, Chinese guidelines, APASL consensus statements, AASLD practice guidance and EASL clinical practice guidelines jointly constitute an international evidence-based framework for standardized diagnosis and treatment of DILI. Despite the absence of uniform recommendations for glycyrrhizin preparations across current international guidelines, available evidence supports their administration for specific subtypes of DILI. Clinical application of these agents requires individualized selection based on patients’ clinical profiles, DILI classification and disease severity. Further high-quality, multicenter international trials are anticipated to refine the global evidence-base for glycyrrhizin preparations.

## 4 Advancing precision therapy: stratified and phenotype-driven application strategies

### 4.1 Precision selection based on clinical phenotypes

Hepatocellular injury-type DILI is the optimal indication for glycyrrhizin preparations (Ma et al., 2023). Beyond their classical anti-inflammatory and membrane-stabilizing effects (Fujisawa et al., 2000; Selyutina and Polyakov, 2019), studies reveal that glycyrrhizin preparations protect hepatocytes, largely through their active metabolite glycyrrhetinic acid (GA) via refined molecular mechanisms. The active metabolite GA alleviates liver injury by inhibiting ferritinophagy and ferroptosis and regulating mitochondrial quality control to preserve hepatocyte function (Jiang et al., 2024). Treatment should follow consensus principles, combining injection and oral sequential therapy after addressing the underlying cause, with dosing guided by prescribing information (Author Anonymous, 2016). Innovations like quercetin-glycyrrhizin nano-gelsdemonstrate superior efficacy in acute liver injury models, significantly reducing serum ALT, AST, and total bilirubin levels compared to traditional formulations (Zhao et al., 2021), pointing to novel developmental directions.

For mixed-type DILI, a combination therapy of glycyrrhizin preparations and ursodeoxycholic acid (UDCA) is recommended (Ma et al., 2023; Author Anonymous, 2016). Glycyrrhizin targets hepatocellular inflammation and necrosis through anti-inflammatory, antioxidant, and hepatoprotective actions (Xia et al., 2025; Wang et al., 2012; Sun et al., 2019); while UDCA addresses cholestasis by promoting bile excretion (Bessone et al., 2024; Bessone et al., 2025). This dual approach synergistically improves biochemical parameters and clinical outcomes. Notably, glycyrrhizin’s anti-fibrotic effects may offer long-term benefits in chronic liver injury, where fibrosis is a hallmark pathology (Wang et al., 2024; Guo et al., 2023).

In cholestatic-type DILI, glycyrrhizin preparations are not recommended as monotherapy but may serve as adjunctive anti-inflammatory agents to UDCA (Ma et al., 2023). Experimental studies show that 18β-glycyrrhetinic acid protects against cholestatic liver injury in bile duct ligation (BDL) models, ameliorating histopathology, serum biomarkers, ductular reaction, oxidative stress, and fibrosis with daily oral administration (Pan et al., 2022). Clinicians should adjust dosages and enhance monitoring in severe cholestasis due to potential pharmacokinetic alterations (Ma et al., 2023).

### 4.2 Severity-based stratified therapy

Tailoring treatment to disease severity is essential for the precision management of DILI. For mild DILI (ALT < 5 × upper limit of normal [ULN]), monotherapy with oral glycyrrhizin preparations is recommended (Ma et al., 2023). Despite the therapeutic efficacy of GA, dose-dependent adverse effects (e.g., pseudoaldosteronism) necessitate careful monitoring, particularly during long-term and/or high-dose administration. Advanced formulations such as GA-loaded nanoplatforms (e.g., UCNPs-GA@Au NCs) enhance hepatic targeting and therapeutic outcomes, offering a promising safety-efficacy balance (Zhao et al., 2021; Meng et al., 2021; Xiao et al., 2024). For moderate DILI (ALT 5–10 × ULN), combination therapy is advised. Intravenous glycyrrhizin preparations serve as the foundational treatment. Meanwhile, adjunctive agents with complementary mechanisms (such as antioxidants and choleretics) are combined, typically using 1-2 such agents (Ma et al., 2023; Author Anonymous, 2016). A randomized trial demonstrated that corticosteroid-glycyrrhizin combination therapyachieves superior efficacy and safety in chronic DILI/HILI, validating this strategy (Wang J.-B. et al., 2022). Severe DILI (ALT > 10 × ULN or with jaundice) requires mandatory aggressive intervention. The treatment regimen should be centered on high-dose intravenous glycyrrhizin preparations, combined with other hepatoprotective agents, and supplemented by robust supportive care (Ma et al., 2023). Severe DILI in special groups (e.g., pregnant women) necessitates immediate, intensive management, reflecting a universal principle for critical cases (Masood et al., 2024). Simultaneously, strict adherence to the recommendations outlined in the Chinese guideline for diagnosis and management of drug-induced liver injury is mandatory. Close monitoring of disease progression should be conducted, and liver transplantation evaluation must be initiated immediately without hesitation once prognostic indicators suggest a poor outcome (Ma et al., 2023). See Table 2.

**TABLE 2:** Systematically outlines the stratified therapeutic strategies for glycyrrhizin preparations in DILI based on severity.

Severity grade	Biochemical criteria	Core therapeutic strategy	Recommended regimen (dosage/route/duration)	Combination therapy	Key considerations	References
Mild	ALT<5 × ULN	Oral glycyrrhizyin preparation	Oral glycyrrhizin preparations (e.g., diammonium glycyrrhizinate or compound glycyrrhizin) as recommended; duration individualized	Not applicable (monotherapy)	Monitor serum potassium levels and blood pressure during treatment	Ma et al. (2023); Zhao et al. (2021); Meng et al. (2021); Xiao et al. (2024)
Moderate	ALT 5–10 × ULN	Intravenous magnesium isoglycyrrhizinate-based hepatoprotective therapy	Magnesium isoglycyrrhizinate 100–200 mg/day by intravenous infusion for 4 weeks	Monotherapy is recommended; combination of two or more hepatoprotective agents is not routinely recommended	Regular monitoring of liver biochemistry, serum potassium, and blood pressure is recommended during therapy	Ma et al. (2023); Wang et al. (2019); Wang et al. (2022b); Author Anonymous (2016)
Severe	ALT > 10 × ULN or accompanied by jaundice	Corticosteroid plus glycyrrhizin combination therapy may be considered in selected patients with chronic DILI agents and supported by vigorous supportive care	Methylprednisolone (stepwise dose reduction over 48 weeks) combined with glycyrrhizin	Emphasizes the combined application of multiple hepatoprotective agents	Management of special populations (e.g., pregnancy) mandates aggressive intervention and close monitoring for poor prognosis, triggering immediate liver transplantation evaluation	Ma et al. (2023); Masood et al. (2024)

### 4.3 Precision management in special populations

DILI exhibits prominent clinical heterogeneity. The underlying diseases, types of culprit drugs, and physiological status of individual patients may all affect its clinical manifestations, disease progression, and therapeutic strategies. Therefore, precise management requires not only attention to special populations but also comprehensive assessment integrating the characteristics of different culprit drugs and individual patient risks. Individualized intervention regimens should be formulated on the basis of timely discontinuation of suspected hepatotoxic drugs. In addition to patients receiving tumor chemotherapy and anti-tuberculosis therapy as well as elderly patients, DILI associated with commonly used clinical agents including antibiotics, nonsteroidal anti-inflammatory drugs (NSAIDs), antiepileptics, statins, and certain cardiovascular medications such as amiodarone also merits clinical attention (Andrade et al., 2019).

#### 4.3.1 Chemotherapy-associated DILI

The prophylactic use of glycyrrhizin preparations holds significant importance for managing chemotherapy-associated DILI, serving as a cornerstone for achieving precision prophylaxis. Studies have shown that the prophylactic administration of magnesium isoglycyrrhizinate before or on the day of chemotherapy can significantly reduce the incidence and severity of DILI in patients with malignant tumors receiving treatment with conventional chemotherapeutic agents (e.g., taxanes, platinum-based drugs, fluoropyrimidines) or novel antineoplastic agents (e.g., molecular targeted drugs, immune checkpoint inhibitors) (Fan et al., 2025). Furthermore, magnesium isoglycyrrhizinate-based combination hepatoprotective regimens exhibit robust clinical efficacy against DILI induced by molecular-targeted therapies and immune checkpoint inhibitors. Key outcomes include significant improvement in liver injury severity grading post-treatment, marked reductions in ALT and AST levels compared to baseline (both *P* < 0.0001). The overall response rates were 69% for ALT normalization and 62% for AST normalization (Duan, 2023).

#### 4.3.2 Anti-tuberculosis drug-induced liver injury (ATB-DILI)

First-line anti-tuberculosis agents such as isoniazid and rifampicin are common causes of DILI (Saukkonen et al., 2006; Liu et al., 2020; Shu et al., 2013). The management of ATB-DILI necessitates individualized strategies. Based on current evidence, glycyrrhizin preparations are not recommended for routine use in the general population to prevent ATB-DILI; they may only be considered on a case-by-case basis in populations at high risk of liver injury. Once acute ATB-DILI occurs, however, glycyrrhizin preparations are recommended as one of the important treatment options. Ammonium glycyrrhizinate or compound glycyrrhizin may be selected for mild-to-moderate injury, while magnesium isoglycyrrhizinate is recommended for moderate-to-severe or mixed injury, to improve liver function parameters and control hepatic inflammatory responses (Zhonghua et al., 2024). The cornerstone of precision management lies in identifying high-risk factors. Patients with the NAT2 slow acetylator phenotype exhibit impaired metabolic capacity for isoniazid, resulting in a significantly elevated risk of DILI. For this population, intensified monitoring or early therapeutic intervention at the onset of liver injury signs should be prioritized (Azuma et al., 2013; Ohno et al., 2000; Zhang et al., 2018).

#### 4.3.3 Management of DILI in elderly patients

In the management of elderly patients with DILI, the coexistence of multiple chronic conditions, polypharmacy, and age-related physiological decline often leads to heightened risks and a significantly higher incidence of adverse outcomes (Ma et al., 2023; Suzuki and MinjunChen, 2025; Weersink et al., 2021; Yu S. et al., 2024). Studies demonstrate that magnesium isoglycyrrhizinate, when added to conventional therapy, significantly enhances treatment efficacy, improves liver function, and exhibits a favorable safety profile (Jin and Qiu, 2015). Its combination with reduced glutathione shows superior advantages in normalizing bilirubin and biliary enzyme parameters (Zhou and Zhou, 2016). Given the prevalence of polypharmacy in elderly patients, concurrent use of two or more potentially hepatotoxic drugs increases the risk of acute liver injury sixfold (Suzuki and MinjunChen, 2025). Clinical practice strongly recommends adopting individualized strategies that prioritize safety and drug-drug interactions. Hepatoprotective agents with established safety profiles (e.g., magnesium isoglycyrrhizinate) should be prioritized, with treatment initiated at low doses and accompanied by close monitoring of liver function, electrolyte levels, and other critical indicators to optimize therapeutic outcomes and ensure medication safety.

In addition, antibiotics, nonsteroidal anti-inflammatory drugs (NSAIDs), antiepileptics, statins, and several cardiovascular agents such as amiodarone also constitute major culprit drugs for DILI in clinical practice. The mechanisms underlying DILI and its clinical manifestations vary considerably among different medications. For instance, antibiotics frequently induce cholestatic or mixed liver injury; antiepileptics are more likely to trigger immune-mediated DILI, whereas amiodarone and statins are mostly associated with long-term medication use. Accordingly, the management of such patients should comply with the general therapeutic principles of DILI. After timely discontinuation of suspected hepatotoxic drugs, individualized treatment shall be administered based on the pattern and severity of liver injury as well as patients’ underlying diseases. Hepatoprotective agents including glycyrrhizin preparations can be applied to ameliorate liver function and suppress inflammatory responses (European association for the study of the liver EASL clinical practice guidelines: Drug-Induced liver injury, 2019).

In summary, precise management should not only rely on clinical classification, disease severity and characteristics of special populations, but also fully take into account the pathological features of different culprit drugs and individual patient risks to achieve truly individualized treatment. At present, the above strategies mainly depend on conventional biochemical indicators (ALT, AST, TBIL) and clinical experience to realize precision medication at the macroscopic level. However, the transition from macroscopic stratification to microscopic individualization requires an in-depth understanding of the intrinsic biological mechanisms underlying the hepatoprotective effects of glycyrrhizin preparations. Recent discoveries regarding gut–liver axis regulation, epigenetic modification and systems pharmacology have not only broadened the comprehension of the classic pharmacology of glycyrrhizins, but also laid a theoretical foundation for elucidating interindividual discrepancies in therapeutic responses among patients with identical DILI subtypes, identifying novel predictive biomarkers of efficacy, and developing next-generation precision intervention regimens. Accordingly, the following section systematically reviews these cutting-edge mechanisms built on the aforementioned clinical evidence, aiming to guide iterative optimization of future precision therapies.

## 5 Exploring cutting-edge mechanisms and novel perspectives

Advances in research technologies have expanded our understanding of the hepatoprotective mechanisms of glycyrrhizin preparations beyond traditional anti-inflammatory and antioxidant properties to broader systems biology dimensions. Recent studies investigating the gut-liver axis, epigenetic regulation, and systems pharmacology networks have provided innovative insights into their multi-target therapeutic profiles, unveiling new horizons for precision applications.

### 5.1 Regulating the gut-liver axis: Discovery of novel mechanisms

**FIGURE 4:**
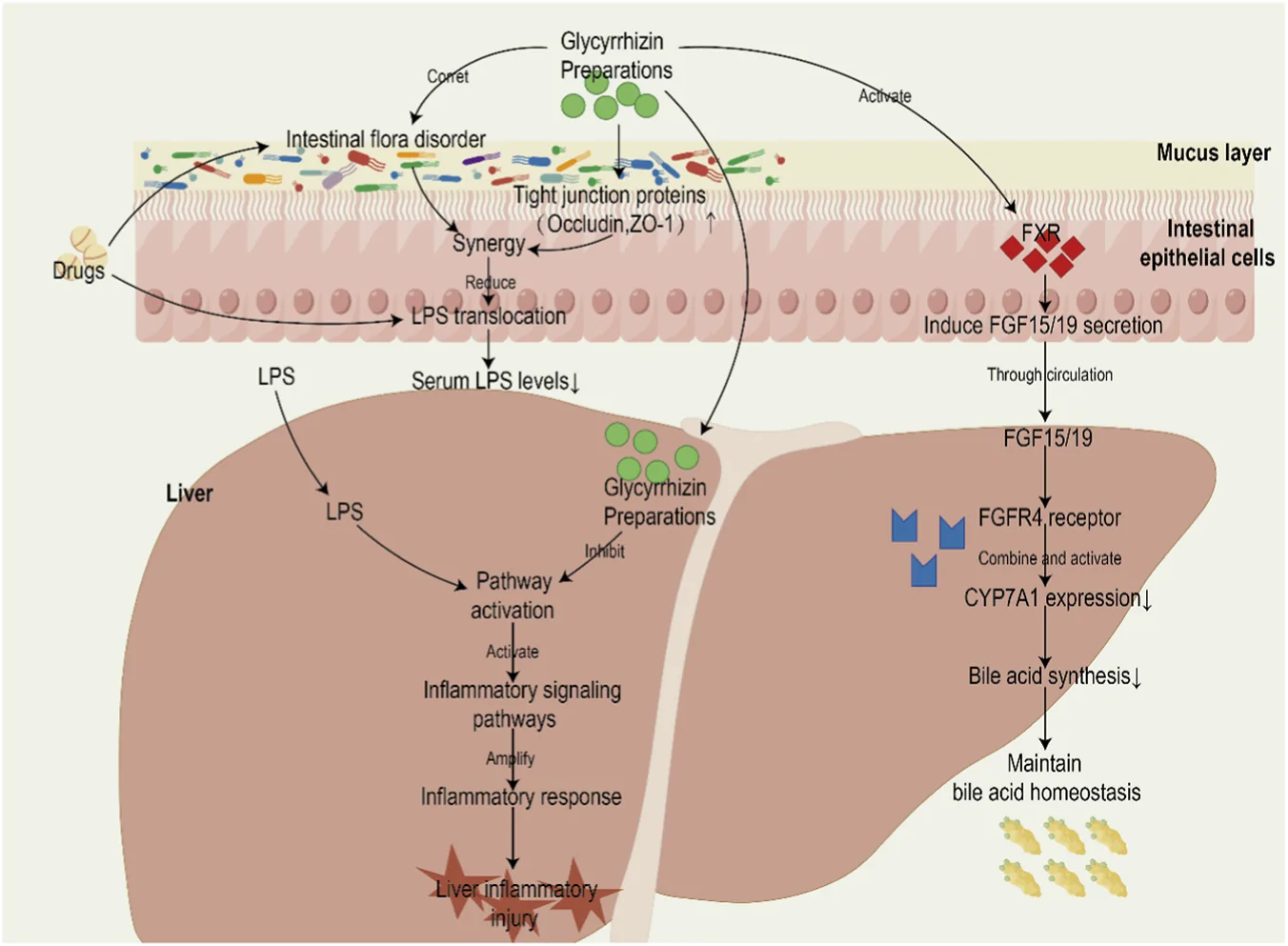
Mechanisms of Glycyrrhizin Preparations in Treating DILI via Modulation of the Gut-Liver Axis. Glycyrrhizin preparations interfere with the gut–liver axis through three key links: ① Modulation of gut microbiota composition: they enrich beneficial bacteria and short-chain fatty acid (SCFA)-producing taxa (e.g., genus *Lactobacillus*), reduce opportunistic pathogens, and restore microbial diversity; ② Enhancement of intestinal barrier function: they upregulate tight junction proteins (occludin, ZO-1, claudin-1), lower intestinal permeability, and thereby attenuate lipopolysaccharide (LPS) translocation into the portal vein circulation; ③ Regulation of bile acid metabolism: they negatively feedback suppress bile acid synthesis via the FXR–FGF15/19–CYP7A1 signaling axis, alleviating hepatotoxicity induced by excessive bile acid accumulation. Collectively, these three processes mitigate LPS/TLR4/NF-κB-mediated hepatic inflammatory responses and confer indirect hepatoprotective activity. Figure was created by the authors.

The gut-liver axis represents the core framework of liver-intestine interactions, where gut microbiota and their metabolites engage in bidirectional communication with the liver through the portal vein circulation (Pabst et al., 2023; Albillos et al., 2020; Tilg et al., 2022). Growing evidence indicates that the progression of DILI is frequently accompanied by gut-liver axis dysfunction, underscoring the therapeutic significance of understanding this bidirectional relationship for DILI management (Chen et al., 2021; Chu et al., 2023). Recent studies reveal that glycyrrhizin preparations exert multi-dimensional modulation of this axis, offering novel mechanistic insights beyond direct hepatocyte protection for DILI treatment (Figure 4).

#### 5.1.1 Modulating gut microbiota composition

The gut microbiota may contribute to DILI through mechanisms such as increased intestinal permeability, disruption of gut metabolite homeostasis, and amplified inflammation and oxidative stress. Alterations in gut microbiota are observed in DILI patients, with dysbiosis identified as a critical promotor of DILI pathogenesis (Li et al., 2024; Tao et al., 2024). Studies have demonstrated that glycyrrhizin preparations and their active metabolites can ameliorate DILI-associated gut microbiota dysbiosis by modulating microbial composition. Animal studies have shown that glycyrrhizin intervention in drug-induced liver injury models increases the abundance of short-chain fatty acid (SCFA)-producing bacteria, such as *Lactobacillus*, while reducing the proportion of opportunistic pathogenic bacteria, thereby improving microbial diversity and metabolic homeostasis (Gong et al., 2022b; Gong, 2022).

Further investigations suggest that these effects may extend beyond alterations in microbial composition and involve a complex regulatory network of “microbiota–metabolites–host signaling”. SCFAs produced by intestinal microbiota, particularly butyrate, can activate G protein-coupled receptors (GPR41/GPR43) and enhance the expression of intestinal tight junction proteins, including zonula occludens-1 (ZO-1), occludin, and claudin-1, thereby preserving intestinal barrier integrity. In addition, SCFAs can regulate the transcription of inflammation-related genes by inhibiting histone deacetylases (HDACs), consequently attenuating intestinal inflammatory responses (Cani et al., 2007; Arpaia et al., 2013).

In DILI animal models, gut microbiota dysbiosis contributes to intestinal barrier disruption, resulting in excessive translocation of lipopolysaccharide (LPS) into the portal circulation. This process activates the TLR4/MyD88/NF-κB signaling pathway in hepatic Kupffer cells, leading to the release of pro-inflammatory cytokines, including TNF-α, IL-1β, and IL-6, and subsequently exacerbating hepatocellular injury (Szabo, 2015). Glycyrrhizin preparations may exert hepatoprotective effects by restoring intestinal barrier function to reduce LPS translocation and directly suppressing TLR4/NF-κB pathway activation, thereby establishing a dual protective mechanism involving “intestinal barrier restoration–inflammatory signaling inhibition”.

Furthermore, regarding bile acid metabolism regulation, the effects of glycyrrhizin on the gut–liver axis may involve the FXR–FGF15/19–CYP7A1 signaling axis. Activation of FXR promotes the secretion of intestinal-derived FGF15/19, which enters the portal circulation and acts on hepatic FGFR4 receptors, thereby feedback-inhibiting CYP7A1 expression and maintaining bile acid homeostasis. Dysregulation of this pathway is closely associated with the development of cholestatic DILI, whereas modulation of FXR-related signaling networks by glycyrrhizin may further alleviate hepatocellular toxicity caused by excessive bile acid accumulation (Gonzalez et al., 2016; Wang H. et al., 2017).

#### 5.1.2 Reducing intestinal permeability and suppressing endotoxin translocation

Impaired intestinal barrier function is a critical step in the translocation of endotoxin (lipopolysaccharide, LPS) to the liver. Once LPS crosses the damaged intestinal barrier into the portal circulation, it activates TLR4 receptors on hepatic Kupffer cells, subsequently triggering downstream inflammatory signaling pathways such as NF-κB, ultimately amplifying hepatic inflammatory responses (Zhang et al., 2022; Gu et al., 2024). This LPS-mediated pathological cascade represents one of the core mechanisms underlying DILI. Glycyrrhizin preparations (e.g., MgIG) can block this pathological process through dual mechanisms to achieve indirect anti-inflammatory and hepatoprotective effects. On one hand, MgIG upregulates the expression of tight junction proteins (e.g., Occludin and ZO-1) in intestinal epithelial cells, enhancing the integrity of the intestinal physical barrier, thereby reducing LPS translocation at its source and ultimately lowering serum LPS levels. On the other hand, even when partial LPS enters the circulation, glycyrrhizin preparations further inhibit the activation of the LPS/TLRs/NF-κB signaling pathway, directly interrupting the LPS-driven inflammatory cascade (Gong et al., 2022b; Gong, 2022). These dual actions collectively constitute the essential mechanism by which glycyrrhizin preparations exert indirect anti-inflammatory and liver-protective effects.

#### 5.1.3 Effects on bile acid metabolism

Bile acids function not only as digestive fluids but also as critical signaling molecules (Z et al., 2025). The maintenance of bile acid metabolism relies on the proper functioning of the farnesoid X receptor (FXR) signaling pathway. FXR is highly expressed in the intestine and liver, and its activation induces the secretion of fibroblast growth factor 15/19 (FGF15/19). After entering systemic circulation, FGF15/19 acts on the FGFR4 receptor in the liver, suppressing the expression of cholesterol 7α-hydroxylase (CYP7A1)—the rate-limiting enzyme in bile acid synthesis—through negative feedback (Jiang et al., 2021; Kim et al., 2016). Studies demonstrate that glycyrrhizin preparations regulate the FXR-FGF15/19 signaling axis, thereby modulating bile acid synthesis and enterohepatic circulation. This regulatory mechanism holds significant therapeutic value for mitigating hepatotoxicity caused by bile acid accumulation, particularly in cholestatic or mixed-type DILI (Yan et al., 2018).

### 5.2 Epigenetic regulatory mechanisms

In recent years, whether the hepatoprotective effects of glycyrrhizin preparations involve the regulation of epigenetic states has emerged as an important research direction, which may provide a potential explanation for some of their long-lasting or sustained therapeutic effects. Among various epigenetic mechanisms, the regulatory role of non-coding RNAs, particularly microRNAs (miRNAs), has gained relatively direct experimental support. miRNAs play critical roles in both physiological and pathological processes in the liver (Klieser et al., 2019; Otsuka et al., 2017). Studies have suggested that glycyrrhizin preparations may modulate the expression profiles of key hepatic miRNAs, among which miR-122 and miR-155 may serve as potential regulatory nodes linking the anti-inflammatory effects of glycyrrhizin with epigenetic regulation (Cong et al., 2023).

miR-122 is one of the most abundant liver-specific miRNAs and is involved in the regulation of hepatocyte differentiation, lipid metabolism, and inflammatory responses. Previous studies have demonstrated that oxidative stress and inflammatory stimuli can lead to decreased miR-122 expression in various liver injury models, whereas restoration of miR-122 levels contributes to the maintenance of hepatocellular homeostasis (Colaianni et al., 2024; Davoodian et al., 2014; Long et al., 2019). Following treatment with glycyrrhizin preparations, the recovery of miR-122 expression suggests that glycyrrhizin may enhance hepatocellular resistance to injury by modulating miRNA-mediated post-transcriptional regulatory networks.

Conversely, glycyrrhizin preparations may regulate inflammatory signaling by suppressing pro-inflammatory miRNAs, such as miR-155. As an important regulator of the NF-κB signaling pathway, aberrant upregulation of miR-155 can promote macrophage activation and inflammatory cytokine production. In inflammatory disease models, glycyrrhizin-related preparations have been shown to reduce miR-155 expression, accompanied by inhibition of NF-κB activation (Zeng et al., 2021; Tili et al., 2007). Therefore, in the context of drug-induced liver injury (DILI), glycyrrhizin may attenuate inflammatory amplification through the potential regulatory axis of “miR-155/NF-κB/TNF-α.” Through the bidirectional regulation of these key miRNAs, glycyrrhizin preparations may restore hepatocellular homeostasis and suppress excessive inflammatory responses at a novel regulatory level.

It is noteworthy that epigenetic regulation extends beyond miRNA-mediated mechanisms (Li, 2021). In addition to the above mechanisms, whether glycyrrhizin preparations exert deeper regulatory effects through histone modifications (e.g., acetylation, methylation) has become a cutting-edge yet under-characterized research direction. Consequently, systematically elucidating the causal relationship between glycyrrhizin and histone modifications will serve as a pivotal step to bridge its traditional pharmacological actions with modern epigenetic theories, advancing its potential for precision medicine. This represents a critical scientific challenge requiring focused investigation in future studies.

### 5.3 Systems pharmacological investigation of signaling pathway networks

Systems pharmacology research provides a holistic perspective for elucidating the multi-target synergistic mechanisms of glycyrrhizin preparations in preventing and treating DILI. By integrating network pharmacology analysis with molecular docking techniques, studies have constructed a “glycyrrhizin-target-pathway-disease” interaction network. Although such analyses often focus on its antiviral and anti-inflammatory properties (e.g., against COVID-19), their revealed core mechanisms remain applicable to DILI contexts. Research demonstrates that its hepatoprotective effects originate not from single-target actions but likely through collaborative regulation of multiple signaling pathways, including PI3K-Akt, MAPK, and Toll-like receptor pathways (Zheng et al., 2021). Specifically, its potential activation of the PI3K-Akt pathway promotes cell survival and inhibits apoptosis (Fu et al., 2025). Its suppression of MAPK pathways (e.g., JNK/p38) correlates with anti-inflammatory and antioxidant stress responses (Li et al., 2018; Fatima et al., 2024). Concurrently, its modulation of the Toll-like receptor/NF-κB signaling axis may further downregulate key inflammatory mediators (Fatima et al., 2024; Hu et al., 2022). These computational predictions, complemented by experimental evidence, collectively outline a multi-pathway interactive network. This system-level analysis offers a comprehensive explanation for the integrative therapeutic characteristics of glycyrrhizin preparations while laying the theoretical foundation for subsequent target validation and precision therapeutic development.

In recent years, international studies have further highlighted that glycyrrhizin preparations and their active metabolites exert typical multi-target and multi-pathway regulatory effects. Their functions cover not only canonical signaling cascades including NF-κB, Nrf2, PI3K/Akt and MAPK, but also a broad spectrum of biological processes such as oxidative stress, immune inflammation, cell apoptosis, metabolic homeostasis and tissue repair. With advances in systems biology, network pharmacology and multi-omics technologies, the integral regulatory network underlying mechanisms of glycyrrhizin preparations of action is being continuously elucidated, laying a novel theoretical foundation for its precision application in DILI and other hepatic disorders (Li X. et al., 2019).

## 6 Challenges and optimization strategies in clinical application

### 6.1 Optimization strategies for risk–benefit balance and patient stratification

Although glycyrrhizin preparations have demonstrated favorable efficacy and an acceptable safety profile in the treatment of DILI, their clinical benefits may be influenced by disease severity, patients’ baseline characteristics, and individual susceptibility to adverse reactions. Therefore, clinical decision-making should not focus solely on therapeutic efficacy. Instead, the anticipated benefits should be carefully balanced against potential risks to achieve individualized risk–benefit optimization. Particular attention should be paid to patients at high risk of pseudohyperaldosteronism (PHA), including older adults and those with renal insufficiency, hypertension, electrolyte disturbances, or concomitant use of diuretics or corticosteroids. In these patients, close safety monitoring is recommended throughout treatment. Based on the current evidence, the optimized clinical application of glycyrrhizin preparations can be established through three key components: baseline risk assessment, patient stratification, and dynamic treatment optimization.

1. Baseline risk assessment. Before treatment initiation, clinicians should comprehensively evaluate patient-related factors, including age, baseline liver and renal function, electrolyte levels, blood pressure, comorbidities, and concomitant medications. This assessment facilitates the identification of patients at high risk for PHA and other treatment-related adverse events and provides a basis for formulation selection and dose optimization.
2. Patient stratification. Patients should be stratified according to the clinical phenotype of DILI (hepatocellular, cholestatic, or mixed type), disease severity (e.g., ALT, AST, and total bilirubin levels, as well as the presence of jaundice), and potential predictive biomarkers. Such stratification helps define treatment priorities and therapeutic goals for different patient subgroups.
3. Dynamic treatment optimization. During therapy, treatment efficacy and safety should be continuously monitored. Early therapeutic response, such as the decline in ALT levels within the first 2 weeks, should be evaluated together with liver function recovery and safety indicators, including serum potassium, blood pressure, and body weight. Dose adjustment, formulation switching, or combination therapy should be considered when necessary to maximize therapeutic efficacy while minimizing the risk of adverse events.

Collectively, these strategies support a closed-loop management pathway consisting of baseline risk assessment, patient stratification, formulation selection, efficacy and safety monitoring, and treatment adjustment. This framework may optimize the risk–benefit profile of glycyrrhizin preparations and further enhance their clinical value in the precision management of DILI. In addition, unified standards for the optimal dosage, treatment timing and therapeutic course of glycyrrhizin preparations in DILI management have not yet been established. Distinct formulations, including magnesium isoglycyrrhizinate, diammonium glycyrrhizinate and compound glycyrrhizin, still exhibit considerable discrepancies in recommended doses, administration modes and total treatment duration. Therefore, clinicians should dynamically optimize therapeutic regimens based on the clinical classification of DILI, disease severity, baseline liver function and early therapeutic response, so as to guarantee clinical efficacy while minimizing safety risks associated with long-term or high-dose medication (Andrade et al., 2019).

### 6.2 Precision management of pseudoaldosteronism (PHA)

Pseudoaldosteronism (PHA), a common adverse effect of glycyrrhizin preparations, results from the inhibition of renal 11β-hydroxysteroid dehydrogenase type 2 (11β-HSD2) by their active components (Monder et al., 1989; Makino, 2021). To achieve precision safety management, the following strategies are recommended.

#### 6.2.1 Risk assessment and formulation selection

To reduce the risk of PHA associated with glycyrrhizin preparations, a comprehensive risk assessment should be conducted before initiating treatment. Prolonged use, high-dose therapy, hypoalbuminemia, elevated direct bilirubin, advanced age, and concurrent use of specific medications (e.g., potassium-wasting diuretics, glucocorticoids) are established risk factors for PHA (Patel et al., 2021; Yoshino et al., 2021; Ishida et al., 2020). For patients with these high-risk characteristics, clinicians should prioritize formulations exhibiting weaker aldosterone-like effects. Studies demonstrate that compared to traditional 18β-glycyrrhizin formulations (e.g., compound glycyrrhizin), 18α-glycyrrhizin magnesium (isoglycyrrhizin) achieves equivalent therapeutic efficacy while significantly reducing the incidence of fluid retention and hypokalemia. This differentiation stems from molecular mechanisms that involve weaker inhibition of 11β-hydroxysteroid dehydrogenase by metabolites of the 18α-isomer, thereby mitigating adverse effects at their source (Chen et al., 2020).

#### 6.2.2 Preventive strategies

For patients with risk factors such as advanced age, hypoalbuminemia, or requiring long-term high-dose therapy, proactive stratified preventive strategies are recommended to significantly reduce PHA incidence. Prophylactic co-administration of aldosterone receptor antagonists (e.g., spironolactone) can competitively block mineralocorticoid receptors at the molecular level, directly counteracting the PHA effects induced by glycyrrhizin preparations and their metabolites (Makino, 2021; Maeda et al., 2008; Sabbadin et al., 2019). Additionally, patients should maintain a potassium-rich diet during treatment, undergo regular monitoring for adverse drug reactions, adjust regimens based on disease progression, and adhere to systematic follow-up protocols (Song and Jiang, 2015; Bi et al., 2024). Crucially, initiating therapy with 18α-isomer formulations like isoglycyrrhizin magnesium, which exhibit inherently lower PHA risk, represents a fundamental source-level risk mitigation strategy (Chen et al., 2020).

#### 6.2.3 Therapeutic drug monitoring (TDM)

TDM serves as an essential tool for optimizing individualized dosing of glycyrrhizin preparations while balancing therapeutic efficacy and safety. When feasible, blood drug concentration monitoring should guide personalized dosing strategies (Ishiwata et al., 2000). Existing studies have established a definite concentration-dependent relationship between plasma concentrations of glycyrrhizin and its active metabolite glycyrrhetinic acid and PHA risk (Meng et al., 2021; Ishida et al., 2020; Manya et al., 2015). Mechanistic pharmacokinetic/pharmacodynamic (PK/PD) models confirm that renal exposure to glycyrrhetinic acid directly drives pseudoaldosterone effects, with serum potassium decline strongly correlating with exposure levels. Sustained trough concentrations exceeding 120 ng/mL (LC-MS/MS) elevate hypokalemia risk by 3.7-fold (Xu et al., 2014). Therefore, in equipped medical centers, TDM-based dose adjustments are critical to maintaining drug concentrations within the therapeutic window, where maximal hepatoprotection coexists with minimal toxicity. Apart from therapeutic drug concentration monitoring, standardized monitoring protocols should be established throughout clinical treatment. It is recommended that liver function indicators including ALT, AST, ALP and TBil be re-examined every 1–2 weeks in the early stage of treatment, accompanied by dynamic monitoring of blood pressure, serum potassium, renal function and body weight changes. For patients receiving long-term therapy or complicated with underlying diseases, the monitoring interval can be appropriately prolonged, and the therapeutic regimen should be promptly adjusted based on efficacy and safety outcomes (Author Anonymous, 2016).

#### 6.2.4 Novel formulation development

To achieve fundamental breakthroughs in the safety profile of glycyrrhizin preparations, the development of next-generation formulations should focus on two strategic approaches that include creating 11β-HSD2-selective inhibitors and engineering glycyrrhizin prodrugs. On one front, rational drug design aims to develop highly selective inhibitors targeting 11β-hydroxysteroid dehydrogenase isoforms, with particular emphasis on minimizing renal 11β-HSD2 inhibition (Chapman et al., 2013). On another front, prodrug strategies are employed to optimize *in vivo* biodistribution, designing derivatives that specifically target the liver and release active components in a site-specific manner, thereby drastically reducing renal electrolyte disruption (Zhao et al., 2021; Liang et al., 2017). These collective efforts strive to completely dissociate the pseudoaldosterone effects while preserving or even enhancing anti-inflammatory and hepatoprotective efficacy, ultimately establishing a safer therapeutic paradigm.

### 6.3 Therapeutic response predictive biomarkers

Moving beyond conventional liver function parameters such as ALT and AST, the identification of predictive biomarkers for therapeutic response is critical to achieving precision management of DILI.

#### 6.3.1 Genetic biomarkers

Individual genetic profiles constitute a pivotal determinant of interpatient variability in drug efficacy and toxicity (Stephens and Andrade, 2020; Urban et al., 2014). Regarding glycyrrhizin preparations, the risk of induced PHA correlates with host metabolic capacity, with studies demonstrating that genetic polymorphisms in key enzymes such as 11β-HSD2 may influence susceptibility to adverse effects like hypokalemia (Wang Y. et al., 2017; Alzahrani et al., 2014). In the broader context of DILI, pharmacogenetic research has established HLA genotypes as critical predictors of drug-specific hepatotoxicity susceptibility (Stephens and Andrade, 2020; Urban et al., 2014). For instance, the HLA-B*35:01 allele has been identified as a genetic risk factor for Polygonum multiflorum-induced DILI (PM-DILI) (Li C. et al., 2019). These discoveries provide actionable tools for etiological diagnosis and risk stratification of DILI. Investigating whether such HLA markers and other genetic signatures can jointly predict both therapeutic response and adverse reaction risks to glycyrrhizin formulations, which thereby enables authentic personalized treatment, represents a crucial frontier for future research. Notably, there remain marked interindividual differences in therapeutic responses to glycyrrhizin preparations among patients. Such heterogeneity in efficacy may be attributed to multiple factors including genetic background, immune status, drug metabolic capacity, underlying diseases and intestinal flora. At present, there are no clinical biomarkers that can stably predict therapeutic response. Accordingly, constructing more precise systems for patient screening and efficacy prediction represents a vital research direction for future investigations (Andrade et al., 2019).

#### 6.3.2 Inflammatory and cell death biomarkers

In the management of DILI with glycyrrhizin formulations, novel serum biomarkers offer a powerful tool for precision-guided dynamic monitoring of therapeutic response. High mobility group box 1 (HMGB1) holds unique value in this context. It serves not only as a crucial damage-associated molecular pattern (DAMP) molecule but also as a recognized therapeutic target of glycyrrhizin, with its serum level reduction directly reflecting the formulation’s anti-inflammatory efficacy (Ma et al., 2023; Yu C. et al., 2024; Niu et al., 2022). Meanwhile, cytokeratin-18 (CK18) and miR-122 enable specific tracking of hepatocyte death processes (apoptosis and necrosis) (Hosack et al., 2023; Korver et al., 2021; Xiong et al., 2025; Humphries and Dear, 2023). Rapid declines in these biomarkers during treatment may facilitate early assessment of hepatic injury resolution and therapeutic effectiveness. The integration of HMGB1 (reflecting targeted anti-inflammatory action) with CK18/miR-122 (indicating cytoprotective effects) establishes a multidimensional therapeutic evaluation system, providing actionable insights to optimize glycyrrhizin-based therapeutic strategies.

#### 6.3.3 Omics-based biomarkers

The construction of predictive models via high-throughput omics technologies represents a cutting-edge approach for proactive therapeutic outcome forecasting. Metabolomics has demonstrated exceptional value in prospective risk stratification, exemplified by PM-DILI studies where pretreatment serum metabolic profiling achieved high accuracy (AUC >0.9) in distinguishing susceptible from tolerant individuals (Cen et al., 2023; Zhang et al., 2020). Proteomics excels in mechanistic elucidation and biomarker discovery, with systematic reviews confirming its capacity to decode protein network perturbations underlying DILI and identify novel blood biomarkers surpassing traditional liver enzymes in precision (Hosack et al., 2023; Kralj et al., 2021). These cross-drug success stories illuminate pathways for precision management of glycyrrhizin therapy. Through unbiased metabolomic/proteomic analysis of characteristic molecular shifts before and after treatment, machine learning models leveraging multidimensional omics signatures may enable pretreatment or early-phase differentiation of probable responders from non-responders, ultimately powering truly personalized therapeutic decisions.

### 6.4 Dosing regimen optimization

Systematic optimization of glycyrrhizin therapeutic strategies is pivotal for enhancing efficacy and safety in DILI management. First, adequate initial dosing intensity should be prioritized to achieve rapid anti-inflammatory effects during the early treatment phase. Mechanistic studies reveal that glycyrrhizin directly binds to key inflammatory mediators like HMGB1 (Yu C. et al., 2024; Niu et al., 2022), and inhibits multiple pro-inflammatory pathways including NF-κB (Author Anonymous, 2016; Gong et al., 2022b; Gong, 2022), thereby effectively interrupting hepatic inflammatory cascades. This vigorous anti-inflammatory intervention provides a critical foundation for hepatocyte regeneration in severe cases (Ma et al., 2023). In addition, patients with DILI often require combined administration of anti-infective agents, anti-tumor drugs, glucocorticoids and other hepatoprotective medications due to their underlying diseases, so potential drug–drug interactions should be fully monitored. Especially for patients receiving long-term combined therapy with diuretics, glucocorticoids or other agents that may disturb electrolyte balance, intensified monitoring of serum potassium and blood pressure is mandatory. Clinicians should timely adjust the dosage or therapeutic regimen of glycyrrhizin preparations according to concomitant medications to reduce the risks of hypokalemia and pseudoaldosteronism (Patel et al., 2021; Yoshino et al., 2021; Ishida et al., 2020). Second, an intravenous-to-oral sequential therapy protocol should be implemented to ensure therapeutic continuity while preventing relapse. This approach not only maintains pharmacological efficacy but also aligns with pharmacoeconomic principles, facilitating seamless transition from hospital care to home-based treatment. Finally, maintaining sufficient treatment duration is essential for sustained therapeutic outcomes (Author Anonymous, 2016). Given that histological recovery typically lags behind biochemical normalization (ALT/AST restoration), treatment should extend beyond transaminase normalization to include consolidation therapy. This phase aims to eliminate residual inflammation, stabilize hepatocyte function, and minimize chronicity/relapse risks (Author Anonymous, 2016; Lowe and John, 2018; Gleeson et al., 2025). The total treatment duration requires comprehensive assessment of baseline liver injury severity, dynamic recovery progression, and complete etiological resolution to achieve truly personalized management. Although randomized controlled trials, meta-analyses and domestic expert consensus have provided favorable evidence-based support for the application of glycyrrhizin preparations in DILI treatment in recent years, high-quality international multicenter randomized controlled trials and long-term follow-up evidence from real-world studies remain relatively scarce. There are also considerable disparities in drug accessibility, clinical practice patterns and guideline recommendations across different countries and regions. Therefore, more real-world studies and long-term safety evaluations need to be conducted in the future to further verify the clinical practicability, long-term efficacy and safety of glycyrrhizin preparations, so as to furnish higher-grade evidence-based foundations for the popularization of precise therapeutic strategies (Andrade et al., 2019; Fontana et al., 2023).

## 7 Conclusion and future perspectives

As foundational agents for the management of drug-induced liver injury (DILI), glycyrrhizin preparations are supported by abundant high-level evidence from evidence-based medicine regarding their efficacy and safety, and have been incorporated into multiple domestic and international clinical guidelines for liver disease diagnosis and treatment. Based on evidence-based medical data, this review primarily establishes a precise therapeutic strategy for glycyrrhizin preparations in DILI. Furthermore, it elaborates on future directions for precision therapy with these agents by integrating emerging mechanisms including the gut–liver axis, epigenetic regulation, and systems pharmacology. With the progressive advancement of precision medicine concepts, the clinical application of glycyrrhizin preparations for DILI is gradually shifting from conventional “empirical medication” toward a stratified, individualized, and precision-oriented paradigm. Clinically, risk-stratified therapeutic strategies—tailored to DILI subtypes, severity, and patient-specific characteristics—are being refined. For hepatocellular injury-type DILI, glycyrrhizin’s anti-inflammatory, enzyme-lowering, and hepatocyte membrane-stabilizing properties establish it as a first-line therapeutic. In mixed or cholestatic DILI, its synergistic potential in combination therapies is increasingly recognized. Notably, glycyrrhizin exhibits consistent safety and adaptability in high-risk populations, including chemotherapy recipients and tuberculosis patients undergoing anti-tuberculosis regimens. Mechanistically, the hepatoprotective actions of glycyrrhizin preparations extend beyond classical pathways (anti-inflammatory, antioxidant, anti-apoptotic) to cutting-edge frontiers. Recent studies highlight its multi-dimensional modulation of the gut-liver axis by reshaping gut microbiota, reducing intestinal permeability, and reprogramming bile acid metabolism to achieve systemic hepatoprotection. Explorations in epigenetics (e.g., histone modifications, non-coding RNA regulation) and systems pharmacology further reveal its genome-wide regulatory potential and multi-target network pharmacology.

While glycyrrhizin preparations demonstrate broad prospects in DILI treatment, their clinical application still faces challenges, particularly adverse effects such as pseudoaldosteronism associated with long-term or high-dose use. Therefore, future research should focus on the following directions. First, actively develop novel glycyrrhizin derivatives or formulations to enhance therapeutic efficacy while reducing adverse reaction risks. Second, integrate multi-omics technologies such as genomics and proteomics to identify biomarkers predicting therapeutic response and risks, thereby establishing personalized medication models. Third, expand research into DILI prevention, particularly investigating their prophylactic value in high-risk drug combinations and genetically susceptible population. Fourth, advance rigorously designed, large-scale prospective studies and real-world research to accumulate high-level clinical evidence and optimize therapeutic protocols. In conclusion, glycyrrhizin remains irreplaceable in DILI management. As precision medicine and mechanistic research evolve, its future application will emphasize individualized risk-benefit profiling, multi-mechanistic integration, and prophylactic-therapeutic synergy, ultimately delivering safer, more precise, and efficacious care for DILI patients.

## Glossary

DILI: Drug-induced liver injury
ALF: acute liver failure
RUCAM: Roussel Uclaf Causality Assessment Method
APAP: acetaminophen
CYP450: cytochrome P450
NAPQI: N-acetyl-p-benzoquinone imine
ROS: reactive oxygen species
APCs: antigen-presenting cells
HLA: human leukocyte antigen
NAT2: N-acetyltransferase 2
GA: glycyrrhetinic acid
PLA2: phospholipase A2
SOD: superoxide dismutase
GSH-Px: glutathione peroxidase
ARE: antioxidant response element
RCT: randomized controlled trial
ALT: alanine aminotransferase
AST: aspartate aminotransferase
HILI: herb-induced liver injury
TBIL: total bilirubin
anti-TB DILI: anti-tuberculosis drug-induced liver injury
MGIG: magnesium isoglycyrrhizinate
APASL: Asian Pacific Association for the Study of the Liver
AASLD: American Association for the Study of Liver Diseases
UDCA: ursodeoxycholic acid
BDL: bile duct ligation
ULN: upper limit of normal
ATB-DILI: anti-tuberculosis drug-induced liver injury
LPS: lipopolysaccharide
FXR: farnesoid X receptor
FGF15/19: fibroblast growth factor 15/19
CYP7A1: cholesterol 7α-hydroxylase
miRNAs: microRNAs
PHA: pseudoaldosteronism
11β-HSD2: 11β-hydroxysteroid dehydrogenase type 2
TDM: therapeutic drug monitoring
PM-DILI: multiflorum-induced DILI
HMGB1: high mobility group box 1
DAMP: damage-associated molecular pattern
CK18: cytokeratin-18
